# Supplementary material for: Improving Soy Sauce Aroma Using High Hydrostatic Pressure and the Preliminary Mechanism
Source: Foods. 2022 Jul 23;11(15):2190. doi: 10.3390/foods11152190 (PMC9330850; doi:10.3390/foods11152190)
Supplement: Supplementary file 1 [file foods-11-02190-s001.zip › foods-1779981-supplementary.pdf]

Table S1 Volatile compounds of the high hydrostatic pressure (HHP)-treated and untreated soy sauces determined by GC-MS.

Compounds	Concentrations (μg/L)									
	Control	HHP200-10	HHP200-30	HHP200-60	HHP400-10	HHP400-30	HHP400-60	HHP600-10	HHP600-30	HHP600-60
Acids										
Acetic acid*	105±8^{f}	146±9^{e}	147±9^{e}	155±9^{e}	185±12^{d}	201±15^{c,d}	218±9^{b,c}	237±10^{a,b}	240±19^{a}	245±17^{a}
2-Methylpropanoic acid	33569±715^{d}	41376±935^{c}	42674±923^{c}	44977±774^{b}	45851±643^{b}	48771±714^{a}	47928±554^{a}	46002±849^{b}	48358±963^{a}	42774±807^{c}
Butanoic acid	–	1254±57^{a}	1302±90^{a}	–	1264±63^{a}	1321±86^{a}	–	–	–	–
2-Methylbutanoic acid	33750±775^{a}	32850±579^{a,b}	33525±782^{a}	33075±926^{a,b}	31725±951^{b,c}	30825±646^{c}	31050±707^{c}	12915±764^{d,e}	11812±777^{e}	13590±823^{d}
3-Methylbutanoic acid	8532.7 ±729^{e}	18684±618^{c}	21060±525^{b}	21438±720^{b}	23652±790^{a}	23760±164^{a}	24408±847^{a}	21600±740^{b}	17388±387^{d}	19116±313^{c}
4-Methylpentanoic acid	–	111±12^{c}	113±13^{c}	123±10^{c}	147±9^{b}	175±8^{a}	183±14^{a}	168±10^{a}	179±14^{a}	190±11^{a}
Hexanoic acid	–	101±3^{c}	112±9^{a,b,c}	115±7^{a,b}	115±6^{a,b}	120±8^{a,b}	124±7^{a}	113±4^{a,b,c}	110±9^{b,c}	117±8^{a,b}
2-Ethylhexanoic acid	24.4±6.3^{e}	79.0±6.6^{d}	85.4±4.5^{d}	–	127±5^{c}	140±3^{b}	144±7^{b}	157±4^{a}	150±9^{a,b}	159±5^{a}
2-Methyl-2-butenoic acid	–	4.71±0.89^{d}	6.35±0.99^{c}	6.92±0.71^{c}	10.5±0.8^{b}	13.5±0.9^{a}	13.2±0.7^{a}	12.8±0.7^{a}	12.6±0.9^{a}	13.4±0.6^{a}
Nonanoic acid	58.6±2.3^{e}	–	68.4±2.5^{c,d}	66.2±5.6^{d,e}	72.5±1.6^{b,c,d}	78.9±3.4^{a,b}	81.2±5.8^{a}	–	75.7±5.7^{a,b,c}	82.3±5.7^{a}
Decanoic acid	–	147±7^{d}	152±3^{c,d}	151±3^{d}	166±10^{b,c}	178±9^{a,b}	175±7^{a,b}	170±3^{a,b}	184±10^{a}	176±11^{a,b}
Benzoic acid	49863±175^{g}	53647±615^{f}	53820±264^{f}	55415±527^{e}	57150±207^{d}	60911±243^{b}	61830±265^{a}	58207±229^{c}	61521±405^{a,b}	56782±539^{d}
Phenylacetic acid	10158±92^{e}	11247±82^{d}	11583±75^{c}	11585±69^{c}	11924±83^{b}	12056±150^{a,b}	12172±167^{a}	12113±70^{a}	12128±102^{a}	12203±92^{a}
Alcohols										
Ethanol*	9525±225^{a,b}	9720±321^{a}	9276±187^{a,b}	8644±166^{d}	9093±249^{b,c}	8820±221^{c,d}	8482±252^{d}	7681±242^{e}	7365±329^{e,f}	7090±253^{f}
1-Propanol	20.5±0.5^{a}	19.6±0.6^{a}	18.5±0.7^{b}	18.3±0.7^{b}	18.1±0.5^{b}	16.8±0.2^{c}	15.8±0.1^{d}	15.6±0.6^{d}	14.5±0.4^{e}	12.5±0.2^{f}
2-Methylpropanol	138±12^{a}	117±14^{b}	110±13^{b,c}	103±11^{b,c,d}	95.1±9.9^{c,d,e}	91.2±8.9^{d,e}	91.0±8.1^{d,e}	82.3±5.0^{e}	84.1±5.6^{e}	77.3±7.4^{e}
1-Butanol	60.6±0.7^{a}	–	51.5±0.6^{b}	47.2±0.8^{c}	43.4±0.4^{d}	41.8±0.4^{e}	41.3±0.4^{e}	36.2±0.4^{f}	34.3±0.7^{g}	32.1±0.9^{h}
2-Butanol	–	10.8±0.1^{d}	10.9±0.1^{d}	11.2±0.2^{c,d}	11.4±0.1^{b,c}	11.8±0.1^{a}	11.8±0.1^{a}	11.3±0.2^{c}	11.9±0.2^{a}	11.7±0.1^{a,b}
2-Methyl-1-butanol	692±17^{a}	654±16^{a,b}	659±24^{a,b}	627±42^{b}	568±35^{c}	521±21^{c,d}	512±31^{d,e}	470±36^{d,e}	464±27^{e}	475±29^{d,e}
3-Methyl-1-butanol	1012±45^{a}	968.3±55.1^{a}	944.5±79.9^{a}	1012±73^{a}	1004±83^{a}	1016±86^{a}	1016±44^{a}	996.7±46.2^{a}	1012±91^{a}	928.3±51.7^{a}
2,3-Butanediol	55.7±3.1^{a}	52.1±3.1^{a,b}	51.6±2.9^{a,b}	51.2±2.9^{a,b}	48.3±2.9^{b,c}	49.2±3.1^{b}	47.5±3.1^{b,c,d}	43.2±2.9^{c,d}	42.4±3.1^{d}	42.0±2.9^{d}
2-Pentanol	9.82±0.41^{b}	10.8±0.1^{a}	10.8±0.2^{a}	10.7±0.2^{a}	10.7±0.1^{a}	10.6±0.1^{a}	10.7±0.3^{a}	10.6±0.1^{a}	10.6±0.2^{a}	10.6±0.3^{a}

Prenol	1.64±0.15[a]	1.56±0.15[a]	1.52±0.04[a]	1.23±0.09[b]	1.03±0.14[c]	0.89±0.09[c,d]	0.74±0.11[d]	0.72±0.06[d]	0.53±0.09[e]	0.40±0.04[e]
4-Methyl-2-pentanol	18.0±0.4[a]	12.6±1.9[b,c]	13.8±2.1[b]	11.2±0.6[c]	–	6.85±0.62[d]	6.41±0.12[d]	4.43±0.41[e]	3.51±0.21[f]	3.02±0.19[g]
2,3-Dimethylpentanol	–	0.35±0.18[c]	1.27±0.16[b]	1.28±0.20[b]	1.32±0.13[b]	1.36±0.16[a,b]	1.44±0.07[a,b]	1.53±0.15[a,b]	1.62±0.16[a]	1.65±0.13[a]
1-Hexanol	1.82±0.18[d]	1.93±0.15[d]	1.67±0.17[d]	2.35±0.19[c]	3.21±0.22[b]	3.69±0.11[a]	3.72±0.16[a]	3.54±0.17[a]	3.79±0.26[a]	–
4-Methyl-1-hexanol	–	–	1.68±0.17[d]	1.73±0.10[c,d]	1.96±0.09[b,c,d]	1.96±0.17[b,c]	2.01±0.14[b,c]	1.89±0.16[b,c,d]	2.03±0.16[b]	2.31±0.15[a]
2-Ethyl-1-hexanol	23.7±4.3[f]	47.7±8.3[c,d]	84.7±7.4[a]	35.4±3.9[d,e,f]	87.1±5.5[a]	96.6±9.2[a]	40.2±8.1[d,e]	71.2±7.5[b]	55.8±7.7[c]	30.4±7.0[e,f]
2-Heptanol	1.27±0.18[d]	2.96±0.34[c]	2.33±0.47[c]	2.41±0.47[c]	4.63±0.49[b]	5.10±0.16[a,b]	5.13±0.34[a,b]	–	5.01±0.49[a,b]	5.32±0.11[a]
6-Methyl-2-heptanol	–	4.71±0.58[b]	4.93±0.47[a,b]	5.01±0.36[a,b]	5.34±0.28[a,b]	5.41±0.37[a,b]	5.46±0.47[a,b]	5.61±0.45[a]	5.64±0.31[a]	5.52±0.20[a]
3-Octanol	6.64±0.71[e]	10.1±0.8[d]	12.9±1.0[c]	13.7±1.2[c]	16.2±0.7[b]	17.5±0.9[a,b]	17.9±0.8[a]	16.3±0.7[b]	16.8±0.4[a,b]	17.9±0.4[a]
1-Octen-3-ol	31.8±1.4[d]	45.6±4.1[c]	49.4±1.2[c]	56.6±2.2[b]	60.3±4.5[b]	66.6±3.7[a]	67.8±1.9[a]	67.5±5.2[a]	67.8±1.8[a]	66.9±5.2[a]
1-Nonanol	0.98±0.08[a]	0.53±0.04[b]	0.51±0.05[b]	0.42±0.03[c,d]	0.44±0.02[c]	0.43±0.03[c]	0.42±0.03[c,d]	0.40±0.02[c,d]	0.37±0.02[c,d]	0.35±0.03[d]
2-Nonanol	–	1.95±0.32[c]	2.31±0.17[c]	2.35±0.25[c]	2.78±0.20[b]	3.17±0.24[a]	3.23±0.15[a]	3.57±0.33[a]	3.59±0.13[a]	3.56±0.13[a]
Benzylalcohol	7.82±0.49[g]	14.8±0.9[f]	15.5±0.7[f]	18.4±0.5[d,e]	19.5±0.4[c,d]	24.9±0.8[a]	23.3±0.6[b]	20.8±0.7[c]	17.7±0.8[e]	17.8±1.0[e]
Methylbenzyl alcohol	–	1.56±0.42[b]	1.73±0.20[a,b]	1.79±0.49[a,b]	2.25±0.43[a,b]	2.34±0.21[a]	2.31±0.14[a]	2.03±0.40[a,b]	2.11±0.42[a,b]	1.95±0.16[a,b]
Phenylethanol*	123±8[a]	111±6[b]	105±5[b,c]	104±6[b,c]	103±6[b,c]	105±7[b,c]	96.2±5.1[c,d]	91.1±9.8[d]	89.4±6.5[d]	87.3±5.2[d]
α-Ethyl phenylethanol	220±11[a]	203±12[a,b]	198±9[b]	190±8[b]	162±11[c]	150±11[c,d]	143±11[d]	121±7[e]	115±10[e]	119±11[e]
1-(2-Butoxyethoxy)-ethanol	–	6.32±0.41[d]	6.79±0.37[b,c,d]	6.95±0.27[b,c,d]	7.51±0.29[b]	8.48±0.46[a]	8.33±0.45[a]	7.23±0.31[b,c]	7.36±0.54[b,c]	6.73±0.41[c,d]
4-Phenyl-3-buten-2-ol	–	15.6±2.2[c]	18.8±2.2[c]	19.8±2.8[c]	25.3±2.8[b]	30.1±2.4[a]	30.8±1.3[a]	33.3±2.7[a]	30.2±2.2[a]	–
Aldehydes										
Acetaldehyde	22.6±0.7[a]	19.5±1.2[b]	17.7±0.8[c]	16.9±1.3[c]	12.1±0.8[d]	10.9±0.6[d,e]	11.0±0.5[d,e]	9.44±0.59[e]	9.01±0.47[e]	8.17±0.58[e]
2-Methylpropanal	–	100±9[b]	–	–	93.5±8.1[b]	96.3±7.6[b]	131±8[a]	–	–	–
2-Methylbutanal	1045±80[d]	1188±52[b,c]	1210±58[a,b]	1232±65[a,b]	1262±46[a,b]	1296±34[a,b]	1311±40[a]	1095±71[c,d]	1091±65[c,d]	1095±73[c,d]
3-Methylbutanal	376±23[b]	416±35[a,b]	429±37[a,b]	422±34[a,b]	433±36[a,b]	451±39[a]	439±28[a,b]	392±32[a,b]	390±30[a,b]	381±27[b]
Benzaldehyde	140±8[a]	52.5±3.7[b]	36.7±2.5[c]	30.2±2.4[c,d]	19.4±1.8[d]	18.3±1.1[d]	23.5±1.2[c,d]	15.4±1.1[d]	16.9±1.7[d]	13.3±0.9[d]
Benzeneacetaldehyde	420±29[d]	463±30[c,d]	528±33[b,c]	545±33[b]	690±43[a]	712±52[a]	651±44[a]	527±31[b,c]	535±46[b]	446±37[d]
3-Furaldehyde	11.2±0.8[d]	16.5±1.4[c]	19.8±0.8[b]	12.1±1.4[d]	20.3±1.5[b]	22.5±1.6[a]	22.8±0.8[a]	23.4±0.7[a]	23.1±1.2[a]	18.7±1.1[b]
Methyl cinnamaldehyde	–	4.32±0.10[d]	5.16±0.19[c]	5.91±0.28[b]	6.31±0.11[b]	7.36±0.31[a]	7.11±0.44[a]	3.90±0.26[d,e]	3.71±0.29[e]	2.52±0.19[f]

Ketones										
Acetone	–	12.7±1.8^{g}	15.5±2.0^{f}	18.6±1.6^{e}	28.4±1.9^{d}	32.2±0.7^{c}	35.9±1.5^{a}	31.1±1.5^{c,d}	32.8±1.2^{b,c}	35.2±1.2^{a,b}
2-Butanone	1.85±0.21^{g}	2.47±0.16^{f}	3.34±0.29^{e}	4.21±0.23^{d}	6.43±0.27^{b,c}	6.78±0.15^{a,b}	6.10±0.22^{c}	6.36±0.24^{b,c}	7.09±0.26^{a}	3.13±0.31^{e}
Acetoin	9.83±0.46^{a}	7.55±0.44^{b}	7.38±0.47^{b}	6.93±0.34^{b}	5.82±0.46^{c}	5.45±0.16^{c,d}	4.94±0.25^{d,e}	4.30±0.51^{e,f}	4.02±0.52^{f}	3.98±0.45^{f}
Methyl isobutyl ketone	–	0.83±0.09^{e}	0.87±0.12^{e}	0.92±0.14^{e}	1.25±0.06^{d}	1.72±0.10^{c}	1.87±0.09^{b,c}	2.11±0.11^{a}	2.01±0.13^{a,b}	2.12±0.12^{a}
2,3-Pentanedione	0.59±0.01^{a}	0.55±0.01^{b}	0.52±0.01^{c}	0.51±0.01^{c}	0.46±0.01^{e}	0.48±0.01^{d}	0.48±0.01^{d}	0.37±0.01^{f}	0.38±0.02^{f}	0.37±0.01^{f}
3-Penten-2-one	–	0.11±0.01^{e}	0.13±0.01^{e}	0.12±0.01^{e}	0.18±0.01^{d}	0.22±0.01^{c}	0.25±0.01^{b,c}	0.29±0.01^{a}	0.27±0.01^{a,b}	0.28±0.01^{a}
5-Methyl-2-hexanone	1.21±0.19^{e}	1.56±0.30^{e}	2.05±0.16^{d}	2.64±0.03^{c}	4.11±0.17^{b}	4.67±0.32^{a}	4.52±0.26^{a}	4.66±0.21^{a}	4.62±0.24^{a}	4.77±0.19^{a}
6-Methyl-2-heptanone	–	1.57±0.11^{b}	1.68±0.09^{b}	1.71±0.10^{b}	2.05±0.10^{a}	2.13±0.11^{a}	2.16±0.11^{a}	2.17±0.09^{a}	2.13±0.11^{a}	2.17±0.10^{a}
3-Octanone	6.50±0.50^{e}	6.93±0.55^{d,e}	7.84±0.52^{c,d}	8.15±0.65^{c}	9.80±0.74^{b}	14.5±1.1^{a}	14.0±0.9^{a}	3.50±0.24^{f}	8.20±0.51^{c}	–
Isophorone	–	1.02±0.09^{f}	1.13±0.04^{f}	1.10±0.06^{f}	2.16±0.10^{b}	2.36±0.14^{a}	1.89±0.08^{c}	1.79±0.04^{c,d}	1.76±0.07^{d}	1.63±0.04^{e}
1-Phenyl-1-propanone	16.9±0.5^{a}	13.2±1.3^{b}	12.7±0.6^{b,c}	12.2±0.8^{b,c,d}	12.4±1.3^{b,c,d}	12.1±1.1^{b,c,d}	11.2±1.3^{c,d,e}	10.7±0.8^{d,e}	10.2±1.0^{e}	9.45±0.51^{e}
2,6,6-Trimethyl-2,4-cycloheptadien-1-one	–	1.57±0.16^{c}	1.59±0.13^{c}	1.63±0.13^{c}	1.96±0.12^{b}	2.18±0.12^{a,b}	2.34±0.17^{a}	2.35±0.15^{a}	2.31±0.14^{a}	2.36±0.16^{a}
3-Hydroxy-3-phenylbutan-2-one	2.84±0.18^{g}	3.37±0.14^{g}	4.79±0.29^{f}	5.51±0.24^{f}	8.01±0.27^{e}	8.31±0.38^{e}	9.17±0.58^{d}	10.1±0.9^{c}	11.8±0.5^{b}	12.9±0.7^{a}
(E)-β-damascenone	–	0.086±0.004^{e}	0.095±0.005^{d,e}	0.097±0.005^{d}	0.119±0.007^{c}	0.176±0.009^{a}	0.159±0.008^{b}	0.065±0.003^{f}	0.074±0.004^{f}	0.070±0.003^{f}
Esters										
Ethyl acetate*	525±24^{a}	506±20^{a,b}	500±24^{a,b}	486±25^{a,b,c}	464±30^{b,c}	445±28^{c,d}	443±28^{c,d}	414±19^{d,e}	401±28^{d,e}	388±22^{e}
Ethyl propanoate	164±9^{a}	158±8^{a}	142±8^{b}	132±7^{b,c}	125±6^{c}	136±8^{b,c}	106±7^{d}	86.0±6.3^{e}	85.6±5.2^{e}	72.6±4.3^{f}
Ethyl lactate	52.1±2.2^{a}	50.4±1.5^{a,b}	47.7±2.2^{b}	42.3±2.1^{c}	38.8±1.4^{d}	31.2±2.1^{e,f}	32.1±1.5^{e}	27.7±2.6^{f,g}	26.5±2.5^{g,h}	23.3±1.6^{h}
Ethyl butanoate	5.04±0.36^{a}	3.32±0.14^{b}	3.02±0.27^{b,c}	2.99±0.10^{c}	1.76±0.18^{d}	1.34±0.19^{e}	1.57±0.08^{d,e}	1.02±0.11^{f}	0.98±0.04^{f}	0.96±0.13^{f}
Ethyl isobutyrate	3.74±0.26^{a}	2.91±0.13^{b}	2.74±0.09^{b}	2.37±0.05^{c}	1.92±0.16^{d}	1.53±0.18^{e}	1.51±0.09^{e}	0.77±0.12^{f}	0.93±0.05^{f}	0.82±0.04^{f}
Ethyl 2-methylbutyrate	23.1±1.1^{a}	20.4±1.0^{b}	16.5±0.9^{c}	16.4±0.9^{c}	10.8±0.7^{d}	7.35±0.41^{e}	7.16±0.34^{e}	6.51±0.23^{e}	6.26±0.35^{e}	5.32±0.27^{f}
Ethyl isovalerate	325±18^{a}	315±18^{a,b}	316±19^{a}	309±19^{a,b,c}	277±19^{c,d}	282±16^{b,c,d}	262±18^{d,e}	238±19^{e,f}	220±17^{f}	217±17^{f}
Ethyl hexanoate	8.55±0.31^{a}	5.49±0.26^{c}	5.62±0.22^{b,c}	4.77±0.24^{d}	4.91±0.18^{d}	5.87±0.22^{b}	2.23±0.13^{e}	1.72±0.11^{f}	1.45±0.10^{f}	1.03±0.05^{g}
Ethyl isohexanoate	4.88±0.29^{a}	3.52±0.21^{b}	3.37±0.18^{b,c}	–	3.15±0.11^{c}	2.76±0.19^{d}	2.58±0.18^{d,e}	2.46±0.17^{d,e}	2.43±0.15^{d,e}	2.41±0.19^{e}

Ethyl 5-methylhexanoate	1.93±0.06^{a}	1.82±0.05^{a,b}	1.78±0.09^{b,c}	1.79±0.07^{b,c}	1.68±0.06^{c,d}	–	1.62±0.05^{d}	1.45±0.09^{e}	1.40±0.06^{e}	1.42±0.06^{e}
Ethyl 2-ethylhexanoate	3.50±0.31^{c}	3.32±0.32^{c}	3.54±0.17^{b,c}	3.63±0.16^{b,c}	4.21±0.31^{a}	4.30±0.35^{a}	4.26±0.18^{a}	4.03±0.36^{a,b}	4.16±0.21^{a}	4.02±0.26^{a,b}
Ethyl heptanoate	3.91±0.05^{a}	1.97±0.09^{b}	1.54±0.07^{c}	–	1.68±0.08^{c}	–	–	–	–	–
Ethyl caprylate	7.05±0.43^{a}	6.57±0.17^{a,b}	6.59±0.17^{a,b}	6.43±0.19^{b}	–	–	–	5.21±0.34^{c}	–	–
Ethyl nonanoate	2.33±0.15^{a}	2.03±0.15^{b}	–	–	1.93±0.13^{b}	2.07±0.12^{b}	–	–	–	–
Ethyl palmitate	13.7±0.8^{a}	11.3±0.7^{b}	10.1±0.6^{c}	9.21±0.45^{d}	6.40±0.44^{f}	4.07±0.21^{i}	4.90±0.34^{h,i}	7.31±0.36^{e}	5.82±0.21^{f,g}	5.25±0.56^{g,h}
Ethyl 9-hexadecenoate	12.3±0.6^{a}	5.58±0.31^{b}	4.46±0.25^{c}	3.15±0.25^{d}	2.70±0.15^{e}	1.08±0.06^{f}	1.21±0.06^{f}	0.94±0.07^{f}	0.82±0.05^{f}	0.91±0.04^{f}
Ethyl oleate	2.02±0.11^{a}	1.64±0.14^{b}	1.51±0.06^{b}	–	–	–	–	–	1.03±0.08^{c}	–
(E)-9-Octadecenoic acid ethyl ester	5.31±0.28^{a}	3.25±0.19^{b}	–	–	2.16±0.15^{c}	–	–	2.05±0.13^{c}	–	–
Ethyl tiglate	1.53±0.07^{a}	1.22±0.08^{b}	0.51±0.07^{c}	–	–	–	–	–	–	–
Ethyl benzoate	210±14^{a}	175±11^{b}	134±12^{c}	130±10^{c}	101±7^{d}	80.6±5.6^{e}	84.2±5.3^{e}	89.5±6.2^{d,e}	91.3±6.1^{d,e}	83.7±4.8^{e}
Ethyl phenylacetate	78.6±4.6^{a}	40.3±3.1^{b}	39.8±2.5^{b}	34.7±2.3^{c}	31.4±1.9^{c,d}	30.8±1.6^{c,d}	24.3±1.8^{e}	28.6±1.5^{d}	20.4±1.6^{e,f}	18.5±1.1^{f}
2-Phenylethyl acetate	503±34^{a}	430±23^{b}	–	–	353±22^{c}	350±29^{c}	338±27^{c}	230±20^{d}	–	–
Ethyl 3-phenylpropionate	4.73±0.19^{a}	2.13±0.09^{b}	1.06±0.07^{c}	–	1.03±0.05^{c}	–	–	–	1.02±0.06^{c}	0.96±0.05^{c}
Butanedioic acid diethyl ester	9.88±0.43^{a}	3.27±0.31^{b}	–	–	2.86±0.23^{b}	–	–	–	–	–
Dimethyl phthalate	2.82±0.15^{d}	3.15±0.13^{c,d}	3.24±0.29^{c}	3.29±0.14^{c}	4.01±0.19^{a,b}	4.12±0.22^{a}	4.05±0.17^{a,b}	4.14±0.11^{a}	3.72±0.19^{b}	3.10±0.11^{c,d}
Methyl benzoate	–	540.2±43.7^{c}	618.2±42.5^{c}	–	953.6±86.4^{b}	1268±86^{a}	1217±90^{a}	–	–	–
Methyl phenylacetate	–	0.87±0.05^{d}	0.75±0.03^{e}	1.06±0.06^{c}	1.20±0.09^{b}	1.35±0.11^{a}	1.38±0.07^{a}	1.19±0.06^{b}	1.02±0.05^{c}	0.42±0.02^{f}
Isobutyl acetate	6.62±0.39^{a}	4.39±0.28^{b}	4.02±0.25^{c}	3.15±0.21^{d}	2.54±0.15^{e}	2.66±0.14^{e}	2.11±0.12^{f}	2.32±0.14^{e,f}	2.05±0.11^{f,g}	1.71±0.10^{g}
Isoamyl acetate	46.3±3.2^{a}	32.3±2.1^{b}	30.1±2.2^{b,c}	28.2±1.2^{c}	22.5±1.7^{d}	21.2±1.5^{d}	20.2±1.3^{d,e}	17.8±1.4^{e,f}	16.4±1.2^{f}	16.9±1.1^{e,f}
Isoamyl lactate	–	15.7±0.9^{d}	16.3±1.0^{d}	16.9±1.1^{d}	27.4±1.4^{b}	31.8±2.3^{a}	–	21.2±1.3^{c}	–	–
Formic acid heptyl ester	–	0.69±0.05^{d}	0.52±0.03^{e}	0.85±0.06^{c}	1.42±0.08^{b}	1.68±0.08^{a}	1.62±0.07^{a}	–	–	–
Butyrolactone	2.16±0.13^{a}	1.42±0.06^{b}	–	–	1.47±0.05^{b}	1.43±0.07^{b}	1.52±0.07^{b}	1.52±0.07^{b}	1.56±0.06^{b}	1.53±0.06^{b}
Furan(one)s										
Furan	–	0.69±0.03^{d,e}	0.76±0.04^{c,d}	0.65±0.03^{e}	0.82±0.05^{b,c}	0.93±0.05^{a}	0.95±0.07^{a}	0.89±0.07^{a,b}	0.96±0.07^{a}	0.97±0.07^{a}
1-(2-Furanyl)-ethanone	–	2.68±0.16^{f}	3.15±0.22^{e}	3.16±0.18^{e}	4.41±0.28^{d}	6.47±0.25^{b,c}	6.15±0.22^{c}	6.90±0.29^{a,b}	7.04±0.32^{a}	7.35±0.39^{a}

1-(2-Furanyl)-1-propanone	13.5±0.6[a]	13.7±0.7[a]	13.7±0.8[a]	14.0±0.7[a]	14.5±0.8[a]	14.5±0.6[a]	14.6±0.6[a]	14.7±0.8[a]	14.7±0.6[a]	14.9±0.8[a]
2,2-Dimethyl-5-isopropyl -furan	1.02±0.08[d]	1.73±0.12[c]	1.66±0.10[c]	1.79±0.11[c]	2.24±0.08[b]	2.17±0.14[b]	2.27±0.12[b]	2.35±0.11[b]	2.67±0.16[a]	2.70±0.16[a]
Benzofuran	0.21±0.02[i]	0.72±0.05[h]	0.89±0.06[g,h]	1.01±0.08[g]	1.79±0.11[f]	2.02±0.12[e]	2.25±0.13[d]	2.67±0.13[b]	2.46±0.18[c]	3.53±0.19[a]
3-Phenylfuran	8.88±0.43[d]	8.84±0.41[d]	8.91±0.46[d]	9.14±0.45[d]	12.5±0.8[c]	14.7±0.9[a,b]	15.1±1.1[a,b]	14.5±1.0[b]	15.9±1.1[a,b]	16.2±1.2[a]
2-Furanmethanol	3609±323[d]	4048±373[c,d]	4220±353[c,d]	4142±328[c,d]	4621±286[b,c]	5148±344[a,b]	5080±292[a,b]	5257±348[a]	5241±351[a]	5460±333[a]
Dihydro-5-pentyl-2(3H) -furanone	–	2.08±0.11[f]	2.63±0.15[e]	2.72±0.15[e]	4.36±0.24[d]	5.00±0.34[c]	5.72±0.17[a,b]	5.04±0.33[c]	5.38±0.22[b,c]	5.95±0.34[a]
HDMF	1244±84[e]	1577±90[b,c,d]	1640±94[a,b,c]	1631±82[a,b,c]	1725±91[a,b]	1772±88[a]	1735±101[a,b]	1677±87[a,b]	1432±105[d]	1497±82[c,d]
HEMF	1860±92[d,e]	1792±88[e]	1851±90[d,e]	1970±91[c,d]	2109±86[b,c]	2329±91[a]	2188±88[a,b]	1810±91[d,e]	1832±92[d,e]	1891±93[d,e]
Phenols										
Phenol	11.8±0.7[b,c]	8.72±0.57[e]	8.45±0.37[e]	8.27±0.59[e]	10.5±0.6[d]	12.3±0.7[a,b]	13.0±0.6[a]	12.5±0.6[a,b]	12.8±0.6[a,b]	11.2±0.5[c,d]
4-Ethylphenol	421±31[a]	361±27[b]	340±24[b]	293±16[c]	285±19[c]	228±17[d]	214±16[d]	175±14[e]	155±11[e]	152±13[e]
3-Tert-butyl-phenol	0.92±0.06[f]	1.13±0.09[d,e]	1.21±0.08[c,d,e]	1.10±0.08[e]	1.26±0.09[b,c,d]	1.32±0.08[a,b,c]	1.35±0.07[a,b,c]	1.33±0.07[a,b,c]	1.39±0.09[a,b]	1.42±0.06[a]
2,4-Di-tert-butylphenol	33.5±2.3[a]	23.5±1.6[c]	24.3±1.7[b,c]	24.7±1.4[b,c]	24.9±1.6[b,c]	15.8±1.6[d]	26.0±2.0[b,c]	26.2±1.9[b,c]	26.3±2.3[b,c]	27.7±2.0[b]
4-Ethylguaiacol	3175±149[a]	2870±137[b]	2620±129[b]	2681±132[b]	2290±182[c]	2051±204[c,d]	2120±130[c]	2016±116[c,d]	1782±166[d]	1790±130[d]
4-Vinylguaiacol	1181±87[a]	945.3±45.2[b]	913.7±71.2[b,c]	819.3±48.0[c,d]	802.5±71.4[d]	747.7±48.2[d,e]	735.3±46.2[d,e]	681.2±43[e,f]	646±49[e,f]	603±34[f]
5-Methylguaiacol	–	0.83±0.06[c]	0.87±0.04[c]	0.93±0.04[b,c]	0.85±0.04[c]	0.62±0.04[d]	1.02±0.05[b]	1.15±0.07[a]	0.91±0.06[b,c]	0.47±0.03[e]
Pyrazines										
Pyrazine	–	0.27±0.03[e]	0.32±0.03[c,d]	0.21±0.01[f]	0.34±0.01[c]	0.28±0.02[d,e]	0.36±0.02[c]	0.46±0.02[b]	0.54±0.03[a]	0.54±0.03[a]
2-Methyl pyrazine	–	4.23±0.26[f]	4.37±0.24[f]	6.58±0.44[e]	8.73±0.58[d]	10.6±0.6[c]	11.0±0.5[c]	12.5±0.7[b]	11.3±0.4[c]	13.6±0.8[a]
2,3-Dimethyl pyrazine	–	0.54±0.03[f]	0.67±0.04[e]	0.68±0.04[e]	0.87±0.05[d]	0.97±0.06[d]	1.09±0.05[c]	1.42±0.05[a]	1.34±0.06[a]	1.21±0.05[b]
2,5-Dimethyl pyrazine	1.32±0.09[e]	1.05±0.06[f]	1.53±0.13[e]	1.46±0.10[e]	2.01±0.11[d]	2.32±0.16[c]	2.41±0.18[b,c]	2.57±0.11[a,b,c]	2.63±0.12[a,b]	2.82±0.15[a]
2,6-Dimethyl pyrazine	–	–	–	6.56±0.43[d]	10.5±0.6[c]	12.8±0.6[b]	13.4±0.7[a,b]	14.0±0.8[a,b]	14.4±0.8[a]	14.6±0.6[a]
2-Isopropyl pyrazine	–	0.36±0.03[f]	0.64±0.03[d,e]	0.42±0.03[f]	0.65±0.04[d]	0.94±0.04[a]	0.84±0.03[b]	0.73±0.04[c]	0.57±0.03[e]	0.82±0.06[b]
Trimethyl pyrazine	2.20±0.13[e]	3.75±0.26[c]	3.08±0.19[d]	3.87±0.23[c]	4.51±0.34[b]	5.32±0.34[a]	4.96±0.27[a,b]	5.43±0.44[a]	5.08±0.25[a,b]	5.42±0.27[a]
2-Ethyl-6-methyl pyrazine	2.90±0.19[f]	3.08±0.25[f]	3.67±0.25[e]	3.14±0.24[f]	4.53±0.29[d]	7.53±0.42[b]	7.42±0.35[b]	7.83±0.42[b]	9.75±0.55[a]	6.32±0.24[c]
2-Ethyl-3,5-dimethyl	0.40±0.02[b]	0.25±0.02[c,d]	0.23±0.01[d]	0.18±0.01[e]	0.28±0.01[c]	0.25±0.02[c,d]	0.23±0.01[d]	0.17±0.01[e]	0.48±0.02[a]	0.42±0.02[b]

pyrazine										
Sulfur-containing compounds										
Disulfide dimethyl	1.40±0.09^{e}	1.76±0.12^{e}	2.94±0.17^{d}	3.05±0.20^{d}	3.86±0.23^{c}	4.75±0.34^{b}	4.90±0.25^{b}	4.97±0.28^{a,b}	5.02±0.34^{a,b}	5.41±0.36^{a}
Dimethyl trisulfide	0.041±0.001^{g}	–	0.072±0.003^{e}	0.064±0.002^{f}	0.088±0.004^{d}	0.108±0.004^{c}	0.121±0.005^{b}	0.133±0.004^{a}	0.139±0.007^{a}	0.141±0.007^{a}
3-Methylthio-1-propanol	513.7±42.5^{f}	1755±112^{e}	1652±105^{e}	1635±109^{e}	2637±164^{b}	3125±185^{a}	2936±148^{a}	2080±132^{d}	2329±153^{c}	2226±138^{c,d}
3-(Methylthio)propanal	189±13^{e}	219±14^{d}	236±15^{b,c,d}	228±16^{c,d}	259±16^{a,b}	277±14^{a}	281±15^{a}	224±13^{d}	254±17^{a,b,c}	239±18^{b,c,d}
2-Hydroxyethyl isobutyl sulfide	14.7±1.4^{c,d}	15.5±1.1^{c}	16.3±1.7^{c}	16.2±1.1^{c}	19.5±1.6^{b}	21.8±1.5^{a,b}	23.6±1.1^{a}	20.5±1.6^{b}	13.2±1.1^{d}	16.5±1.0^{c}
Ethyl 3-(methylthio) propionate	16.8±1.1^{a}	16.5±1.2^{a}	14.1±1.0^{b}	10.3±0.7^{c}	17.8±1.3^{a}	17.6±1.4^{a}	17.5±1.3^{a}	9.03±0.45^{c}	8.75±0.32^{c,d}	7.20±0.34^{d}
3-(Methylthio) propyl acetate	5.95±0.27^{a}	5.91±0.18^{a}	4.76±0.24^{b}	4.53±0.23^{b}	3.71±0.26^{c}	2.98±0.13^{d}	2.75±0.12^{d,e}	2.32±0.11^{f}	2.01±0.16^{g}	1.92±0.11^{g}
Others										
2-Acetylpyrroline	0.83±0.05^{e}	0.89±0.04^{e}	0.90±0.05^{e}	0.92±0.05^{d,e}	1.00±0.05^{d}	1.19±0.05^{c}	1.14±0.05^{c}	1.29±0.06^{b}	1.44±0.06^{a}	1.40±0.05^{a}
3-Methyl-2-pyrrolidinone	2.57±0.15^{a}	2.63±0.17^{a}	2.66±0.17^{a}	2.67±0.14^{a}	2.74±0.21^{a}	2.79±0.22^{a}	2.79±0.18^{a}	2.81±0.22^{a}	2.78±0.16^{a}	2.79±0.23^{a}
Maltol	13.5±1.1^{c}	16.7±1.2^{a,b}	17.1±1.2^{a,b}	17.2±1.2^{a,b}	17.8±1.3^{a}	18.0±1.4^{a}	17.4±1.2^{a}	15.1±1.3^{b,c}	13.0±1.0^{c}	–
Naphthalene	5.08±0.38^{a}	5.42±0.36^{a}	5.35±0.49^{a}	5.61±0.42^{a}	5.27±0.38^{a}	5.36±0.47^{a}	5.18±0.44^{a}	4.97±0.37^{a}	5.26±0.32^{a}	5.41±0.42^{a}
Dodecane	1.47±0.08^{a,b}	1.53±0.06^{a}	1.46±0.07^{a,b}	1.44±0.07^{a,b}	1.02±0.07^{e}	1.38±0.06^{b,c}	1.35±0.06^{b,c,d}	1.36±0.09^{b,c,d}	1.27±0.07^{c,d}	1.22±0.09^{d}
Hexadecane	2.48±0.15^{a}	2.55±0.18^{a}	2.53±0.13^{a}	2.58±0.16^{a}	2.62±0.17^{a}	2.67±0.15^{a}	2.58±0.16^{a}	2.69±0.15^{a}	2.74±0.14^{a}	2.49±0.13^{a}

* The unit of compound concentration is mg/L. $^{-}$ not detected in GC-MS. $^{a\text{–}j}$ Different letters in the same row indicate the significant differences ($p < 0.05$).
